# Molecular Characterization of Complement Component 3 (C3) in the Pearl Oyster *Pinctada fucata* Improves Our Understanding of the Primitive Complement System in Bivalve

**DOI:** 10.3389/fimmu.2021.652805

**Published:** 2021-04-19

**Authors:** Zhongliang Wang, Xueru Liang, Guiying Li, Bai Liufu, Kaiqi Lin, Jinfeng Li, Jing Wang, Bei Wang

**Affiliations:** ^1^ College of Fisheries, Guangdong Ocean University, Zhanjiang, China; ^2^ Guangdong Provincial Key Laboratory of Pathogenic Biology and Epidemiology for Aquatic Economic Animals, Guangdong Ocean University, Zhanjiang, China

**Keywords:** *Pinctada fucata*, innate immunity, complement component 3, C3, Luciferase reporter assays, phagocytosis, flow cytometry

## Abstract

As the central component in the complement system, complement component 3 (C3) plays essential roles in both the innate and adaptive immune responses. Here, a C3 gene (designated as *pf-C3*) was obtained from the pearl oyster *Pinctada fucata* by RT-PCR and rapid amplification of cDNA ends (RACE). The *pf-C3* cDNA consists of 5,634 bp with an open reading frame (ORF) of 5,193 bp encoding a protein of 1,730 amino acids with a 19 residue signal peptide. The deduced pf-C3 protein possessed the characteristic structural features present in its homologs and contained the A2M_N_2, ANATO, A2M, A2M_comp, A2M_recep, and C345C domains, as well as the C3 convertase cleavage site, thioester motif, and conserved Cys, His, and Glu residues. Phylogenetic analysis revealed that pf-C3 is closely related to the C3s from other mollusks. *Pf-C3* mRNA was expressed in all examined tissues including gill, digestive gland, adductor muscle, mantle and foot, while the highest expression was found in the digestive gland. Following the challenge with *Vibrio alginolyticus*, *pf-C3* expression was significantly induced in hemocytes. Luciferase reporter assays indicated that pf-C3a could activate the NF-*κ*B signal pathway in HEK293T cells. Further knockdown of *pf-C3* by specific siRNA could significantly reduce the phagocytosis of *V. alginolyticus* by hemocytes *in vitro*. These results would help increase understanding of the function of C3 in the invertebrate immune system and therefore provide new insights into the roles of the primitive complement system in invertebrates.

## Introduction

The innate immune system in all metazoans is an evolutionarily ancient form, which offers the non-specific defense and exerts an important role in the first-line defense to resist foreign invaders or pathogens. The complement system comprises over 30 soluble plasma proteins and certain membrane-associated proteins (1), which represents a main mechanism of innate immunity, exerts a vital part in the anti-infectious agent adaptive and innate immune responses, and is involved in numerous physiopathological processes. Typically, the complement system mainly functions to regulate inflammation, cytolysis, phagocytosis, immune complex solubilization, apoptotic cell elimination, and humoral immune response enhancement. Additionally, the activation of the complement system also contributes significantly to the adaptive immune responses (2, 3).

The complement system in mammals is activated through series of restricted proteolytic cascades *via* three independent pathways, namely, the classical, lectin, and alternative pathways. The above three pathways are combined during the complement component 3 (C3) proteolytic activation process; meanwhile, activating the C3 fragments induces certain functions to resist pathogens, thus facilitating immune responses (1). These mechanisms comprise opsonization of C3b coated non-self particles (4, 5), and inflammation develops by means of C3a anaphylatoxin together with C5 component activation, finally inducing terminal complement proteins that constitute membrane attack complex (MAC) (4, 6).

Recently, a large number of complement-like molecules were identified in bivalves. For example, C3-like and factor B-like (Bf-like) molecules in the clam *Ruditapes decussatus* and *Sinonovacula constricta* (4, 7) and C3, C1q-domain-containing proteins (C1qDC), collectin-like proteins, fibrinogen-related proteins (FREP), serine proteases, and complement receptor-like proteins in the oyster *Crassostrea gigas* (2). The identification of complement components indicated the existence of a multi-component complement system in bivalves. Considering the presence of C3-like and Bf-like molecules in clams, an alternative pathway might be dedicated to the activation of the complement system in bivalves. However, the distinctive structure (composed of two CCPs which is one less than in vertebrates) and inhibition of the expression by live bacteria make *R. decussatus* Bf-like a novel protein related to the Bf/C2 family proteins, and whether it may participate in the activation of the complement system requires further validation. In the oyster, although the potential complement-related molecules such as C1qDCs, CTLs, and FREP families are all greatly expanded, the lack of typical Bf/C2/MASP or MBL/ficolin molecules indicates that the conventional alternative pathway and lectin pathway either do not exist in the oyster or the complement activation pathways in the oyster were likely to have undergone major changes during evolution (2). The complement system in the oyster functions probably by different mechanisms from that reported in ascidian *Ciona intestinalis* and amphioxus *Branchiostoma floridae*, in which a number of genes encoding components in the lectin and alternative activation pathways were found from genomic analysis (2, 8, 9).

Complement C3, the *β*2 glycoprotein that consists of the *α* and *β* peptide chains, is one of the thioester-containing protein (TEP) superfamily members (10). In comparison with other complement components, C3 is found at the highest proportion in serum and plays a role of a pivotal part of the complement system (10). Since C3 was first identified in 1912, its roles in immune response have attracted much attention and have been studied mainly in mammals and other higher vertebrate species. As the core of an ancient immune defense system, C3s or C3 homologs have been reported in a variety of invertebrates, such as urochordates, cephalochordates, echinoderms, arthropods, or cnidaria over the last several years (1, 11–16). Recently, C3 homologs have also been identified from the mollusks, including carpet-shell clam, razor clam, oyster, common mussel, owl limpet, and Hawaiian bobtail squid (2, 4, 10, 17–20). In several species of bivalve, the up-regulated expression of C3s upon lipopolysaccharides (LPS) and bacterial exposure suggested that, the invertebrate C3s was able to identify the pathogenic microorganisms (2, 3, 10, 17, 21). Moreover, C3 from razor clam was found to control hemolytic activity, which suggested that C3 is likely to control the mechanisms of membrane rupture that the complement system uses in shellfish (17). To date, however, the information on C3 molecules in mollusks is still fragmentary; especially available information is deficient regarding the physiological functions. Whether the C3s in mollusk species play roles similar to vertebrate C3s remains largely unknown.

The pearl oyster, *Pinctada fucata*, is the most important species cultured widely for production of marine pearls in the coastal provinces of South China (22). It was successfully hatched in 1965; since then, it has become a critical economic resource. But the cultivated pearl oysters, especially for the young, operated, and mother oysters, experience high death rates recently (22, 23). This severely reduces the number of maricultured pearls and destroys the pearl oyster resources (22). In this context, to elucidate the immune defense mechanisms of the pearl oyster may contribute to the development of novel management strategies for diseases control and the long-term sustainability of the pearl industry. In the present study, the complement C3 gene in *P. fucata* (*pf-C3*) was cloned and characterized for the first time, and the expression patterns including tissue-specific expression and temporal expression after being challenged by *V. alginolyticus* were also investigated. Additionally, dual-luciferase reporter assays were performed to evaluate the activation of NF-*κ*B signaling transduction by *pf-C3a*, and RNA interference (RNAi) assay was also conducted to investigate the regulation of phagocytosis against *V. alginolyticus* by pf-C3. The work might provide a clue for further studies on bivalve C3 and expand the horizon to better understand the functional evolution of the ancient complement system.

## Materials and Methods

### Animals, Immune Challenge and Isolation of Total RNA

Pearl oysters, *P. fucata*, averaging 65 mm in shell length, were purchased from a pearl oyster farm in Zhanjiang, Guangdong Province, China and maintained in tanks containing 80 L aerated sand-filtered seawater at 25°C, and fed daily with *Spirulina* powder for 1 week prior to processing.

For immune challenge, healthy pearl oysters were randomly divided into two groups. Each pearl oyster of the challenged group was injected with 5 × 10^7^ CFU/ml of *V. alginolyticus* resuspended in 100 µl PBS (Phosphate Buffered Saline) into the adductor muscles, and each those of the control group was injected with 100 μl PBS. At each time point (0, 2, 4, 8, 12, 24, and 48 h) post-injection, hemolymph (about 0.5 ml per individual) was collected from the control group and challenged group using a syringe from the adductor muscles and immediately centrifuged at 800×g, 4°C for 10 min to harvest the hemocytes (22, 24). For tissue-specific expression analysis, different tissues (digestive gland, gill, adductor muscle, mantle, and foot) and hemolymph were sampled from the control and challenged group at 4 h post-injection. Pearl oysters of each experimental group were divided into three replicates with twenty individuals and fed in three tanks. Six pearl oysters (two individuals from each tank) were randomly sampled from each experimental group at each time point, and hemolymph and tissues were mixed together with equal amounts as one sample (22, 24).

For total RNA isolation, the hemocyte pellets and tissues were placed into the TRIzol reagent (Invitrogen, USA) and further processed according to the protocol provided. Isolated total RNA was stored at −80°C and used for cDNA synthesis or real-time PCR described below.

### Determination of the Full-Length *pf-C3* cDNA Sequence

By scanning the *P. fucata* genome database (25), one predicted mRNA sequence of 1,599 bp homologous to the C3 of *C. gigas* (GenBank accession no.: XP_034335787) was found, and this C3 sequence was selected for further cloning of the full-length cDNA of *pf-C3*. Based on the sequence, primers were designed to amplify the rest cDNA sequences of *pf-C3* gene (**Table 1**). Following, the 3′- and 5′-ends of *pf-C3* were obtained employing a 5′/3′ RACE (Rapid Amplification of cDNA Ends) kit (2nd Generation, Roche, Germany) as specified by the manufacturer.

**Table 1:** Primers used in the present study.

Primers	Sequences (5′–3′)	Use
C3-RT1	GTTATTGGTTGTAGGGAGGA	For 5´ RACE
C3-RT2	ATCTCTACGGAAAATCTCG	
C3-R3	TCATTCAGAGGCAAACTTGGGTCCGT	
C3-R4	GGCAAACTTGGGTCCGTGCTCTGT	
C3-F1	TTCAACTGTTGTGCATTCCGCGATAA	For 3´ RACE
C3-F2	AGGACCAGGTAACATACGTGTTGGCGAT	
qPCR-C3-F	CAAGATTATCCCCAGCGGCA	For real-time PCR
qPCR-C3-R	ACCTGTCGGTCCTCTTCGTCAC	
qPCR-actin-F	TGGTATGGGACAGAAGGAC	
qPCR-actin-R	GACAATGCCGTGCTCAAT	

The obtained PCR products were separated on 1% agarose gel, and then gel-purified with the QIAquick Gel Extraction Kit (Qiagen, Germany). The purified PCR products were ligated into pMD18-T vector (Takara, Japan) and transformed into competent *Escherichia coli* cells. The recombinants were identified through blue/white color selection in ampicillin-containing LB plates and screened with M13 primers. The positive clones were sequenced by Sangon Co., Ltd. (Shanghai, China). At least three different clones of each amplicon were sequenced to eliminate PCR artifacts.

### Bioinformatics Analysis

The similarity analysis of nucleotide and protein sequences was carried out by using BLAST program at NCBI (http://blast.ncbi.nlm.nih.gov/Blast.cgi). The functional domains in the deduced amino acid sequence were predicted using Conserved Domains at NCBI (https://www.ncbi.nlm.nih.gov/Structure/cdd/wrpsb.cgi) and SMART (http://smart.embl-heidelberg.de/). The potential N-glycosylation sites and signal peptide sequences were predicted using NetNGlyc 1.0 Server (http://www.cbs.dtu.dk/services/NetNGlyc/) and SignalP-5.0 Server (http://www.cbs.dtu.dk/services/SignalP/), respectively. Multiple alignments of the amino acid sequences were performed with the Clustal Omega Multiple Alignment program (https://www.ebi.ac.uk/Tools/msa/clustalo/) and optimized manually. Based on the sequences of *pf-C3* and other known C3 homologs, a phylogenetic tree was constructed by the Maximum Likelihood (ML) method using the MEGA X software (version 10.2.4). The tool of “Find Best DNA/Protein Models” was used and “WAG+G+I+F” model was chosen to construct the phylogenetic tree. The reliability of the tree was tested by bootstrapping using 1,000 replications (sequences used to construct phylogenetic tree with the abbreviation and GenBank accession number were listed in the legend of **Figure 2**).

### Quantification of *pf-C3* Expression by Real-Time PCR

Real-time PCR was applied to evaluate the mRNA expression level of *pf-C3* in hemocytes as well as in different tissues after *V. alginolyticus* challenge. First-strand cDNA synthesis was carried out based on PrimeScript Reverse Transcriptase (Takara, Japan) using total RNA treated with DNase I (NEB, USA).

Two specific *pf-C3*-specific primers, qPCR-C3-F and qPCR-C3-R (**Table 1**), were used to amplify a product of 131 bp, and the PCR product was sequenced to verify the specificity of real-time PCR. A pair of *β*-actin primers (**Table 1**), based on sequence from GenBank (accession nos.: EU726273), was used to amplify fragments of 94 bp served as endogenous controls for amount of cDNA.

The real-time PCR assay was carried out in a Bio-Rad iQ™5 multicolor real-time PCR detection system (Bio-Rad, USA). All samples were analyzed in triplicate. The amplifications were performed in a 20 μl reaction volume containing 10 μl of 2× SYBR Premix Ex *Taq* (Takara, Japan), 0.4 μl of each gene-specific primer (10 mM), 2 μl of template cDNA (diluted to 1:60), and 7.2 μl of PCR-grade water. The thermal profile for real- time PCR was 94°C for 30 s followed by 40 cycles of 94°C for 5 s, 59°C for 15 s and 72°C for 10 s. Dissociation curve analysis of amplification products was performed at the end of each PCR reaction. To maintain consistency, the baseline was set automatically by the software. The comparative Ct method was used to analyze the expression level of *pf-C3*.

### Dual-Luciferase Reporter Assay

The cDNA fragment encoding pf-C3a (corresponding to amino acid residues 654 to 744 of pf-C3) was amplified by PCR with primers containing HindIII and XhoI restriction enzyme cutting sites. The PCR products were digested with HindIII and XhoI and ligated and subcloned into the plasmid expression vector pcDNA3.1 digested by the corresponding restriction enzymes. The plasmid constructed was verified by sequencing and designated as pcDNA3.1-C3a.

HEK293T cells were cultured in Dulbecco’s Modified Eagle Medium (DMEM, Gibco, USA), supplemented with 10% fetal bovine serum (FBS, Invitrogen, USA), 2mM L-glutamine, 100 units/ml penicillin and 100 mg/ml streptomycin in TC plate in humidified incubator with 5% CO_2_ at 37°C. pcDNA3.1-C3a or empty vector pcDNA3.1 along with pNF-*κ*B luciferase reporter plasmid (Clontech, USA) or pGL3-Basic luciferase reporter plasmid were transfected into HEK293T cells using Lipofectamine 2000 Reagent (Invitrogen, USA) for 48 h according to the manufacturer’s recommendation. pRL-TK renilla luciferase plasmid (Promega, USA) was used as an internal control. All assays were performed with three independent transfections.

Luciferase activity of total cell lysates was measured using the Dual-Luciferase^®^ Reporter Assay System (Promega, USA) as described in the manufacturer’s instructions. The relative luciferase activities of cells treated by recombinant proteins were normalized to the luciferase activity produced by pRL-TK plasmid. The induction was expressed as fold changes by comparing the luciferase activities of recombinant vector inducted cells with that of empty vector inducted cells.

### siRNA-Mediated Knockdown of Gene Expression

Three fragments of short-interfering RNAs (siRNA): si-83 (5′-CCAGTAAACTACGTTACAA-3′), si-1535 (5′-GCATATTAATCCAGCCTTT-3′), si-1745 (5′-GCAGGAGAGCTGACATTAA-3′) were designed by BLOCK-iT™ RNAi Designer (Invitrogen, USA). Another siRNA (negative control, NC) without targeting any unigenes in transcriptome data was served as negative control. C3-siRNA was synthesized using an *in vitro* transcription T7 Kit (Takara, Japan) under RNase-free conditions, and the primers for double-strand Oligo DNA annealing reaction were synthesized by Sangon Co., Ltd. (Shanghai, China) and designed according to the manufacturer’s instructions.

The siRNA was diluted using phosphate-buffered saline (PBS). For the gene knockdown experiment, 100 μl of PBS containing 15 μg of siRNA was injected into the adductor muscle of *P. fucata* and then injected twice after 12 h to compensate for RNA degradation. Every set contained at least three pearl oysters as biological replicates. The relative expression levels of *pf-C3* after RNA interference were detected by Real-time PCR to select the most efficient siRNA for further experiment. The variation of *pf-C3* expression after siRNA treatment was determined by real-time PCR, referring to the expression of the PBS-treated *P. fucata*.

### Fluorescent Labeling of *V. alginolyticus*

Bacteria *V. alginolyticus* from a stationary-phase culture were heat-killed at 75°C for 10 min. After washing twice with 0.1 M Na_2_CO_3_–NaHCO_3_ (volume ratio was 1:1, pH 9.8), the bacteria were incubated in 0.1 M Na_2_CO_3_–NaHCO_3_ containing 0.1 mg/ml fluorescein isothiocyanate (FITC) (Sigma, USA) at 25°C for 2 h with gentle stirring. Subsequently, the labeled bacteria were washed with PBS until the supernatant was free of visible FITC and resuspended in PBS.

### Phagocytosis Assay

Hemolymph from the siRNA treated group and the control group was withdrawn through a disposable syringe (three pearl oysters were randomly sampled from each group) and mixed with equal volume anticoagulant. The mixture was centrifuged at 800×g and 4°C for 10 min to harvest hemocytes. The hemocytes were suspended in PBS, counted, and adjusted with PBS to a cell density of 3–5 × 10^6^ cells/ml, and separated into three tubes. Then, 100μl of FITC-labeled bacterial suspension was added to the hemocytes and incubated in the dark for 1 h with gentle stirring every 5 min. Thereafter, these cells were washed thrice with PBS to remove excess bacteria, and 250 μl 4% paraformaldehyde (PFA) was added to the mixture for flow cytometry analysis. All data were repeated three times to ensure the accuracy of the analysis.

### Statistical Analysis

All data obtained from this study were expressed as the means ± SD (standard deviation) of three repeated experiments and subjected to a one-way ANOVA or Student’s t-test to determine differences among the treatments. Differences were considered statistically significant at *P <*0.05. Statistical analysis was performed using SPSS 22.0 for Windows.

## Results

### *pf-C3* Cloning and Molecular Characterization

Based on the EST of 1,599 bp identified from the genome database of *P. fucata*, the cDNA fragments covering the full-length of *pf-C3* was cloned by using 3′ RACE and 5′ RACE. The complete cDNA of *pf-C3* is 5,634 bp, containing a 5′ untranslated region (UTR) of 85 bp, a 3′ UTR of 356 bp and an opening reading frame (ORF) of 5,193 bp, which encodes a protein of 1,730 amino acid residues (GenBank accession no. MT502525) (**Supplementary Figure 1**). The theoretical mass of the whole mature pf-C3 was calculated to be 196.80 kDa with an isoelectric point 5.68. The presumptive signal peptide is formed by the first 19 residues as predicted by the SignalP-5.0 Server, indicating that it is a secreted protein (**Supplementary Figure 1**). Eleven potential N-glycosylation sites of Pf-C3 were identified (N^113^, N^248^, N^292^, N^385^, N^404^, N^467^, N^533^, N^686^, N^705^, N^1344^, and N^1364^) (**Supplementary Figure 1**).

**Figure 1:**
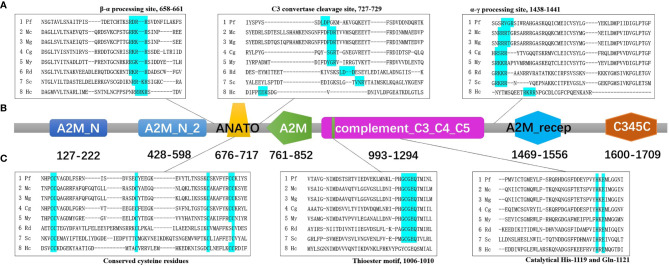
The predicted functional domains and conserved sites of pf-C3. **(A)** Multiple alignments of partial amino sequences of selected C3 proteins from bivalves to show the vital functional loci of *β*–*α* processing site, C3 convertase cleavage site and *α*–*γ* processing site. **(B)** The predicted functional domains of pf-C3. A2M_N, A2M_N_2, ANATO, A2M, complement_C3_C4_C5, A2M_recep and C345C domain were marked with colors sequentially. **(C)** Multiple alignments of partial amino sequences of selected C3 proteins from bivalves to show the vital functional loci of thioester motif and conserved Cys, His, Glu residues. The abbreviations of species names and GenBank accession number are: Pf, *Pinctada fucata* C3 (MT502525); Mc, *Mytilus coruscus* C3 (MG197986); Mg, *Mytilus galloprovincialis* C3 (AJQ21542); Cg, *Crassostrea gigas* C3 (NP_001292308); My, *Mizuhopecten yessoensis* C3 (OWF37722); Rd, *Ruditapes decussatus* C3 (ACN37845); Sc, *Sinonovacula constricta* C3 (ANI85912); Hc, *Hyriopsis cumingii* C3 (MK648113).

The result of predicted functional domains and conserved sites analysis of Pf-C3 revealed the presence of seven conserved domains including A2M_N (*α*2-macroglobulin family N-terminal region, residues 127–222), A2M_N_2 (residues 428–598), ANATO (anaphylatoxin-like domain, residues 676–717), A2M (*α*2-macroglobulin family domain, residues 761–852), complement_C3_C4_C5 (residues 993–1294), A2M_recep (*α*2-macroglobulin receptor, residues 1,469–1,556), and the C345C domain (residues 1,600–1,709) (**Figure 1**).

Multiple alignment of amino acid sequence of pf-C3 with other known C3 sequences from mollusks using Clustal Omega (https://www.ebi.ac.uk/Tools/msa/clustalo/) indicated that the *β*–*α* junction site (RDRR) and *α*–*γ* junction site (RVGR) are located at amino acids 658–661 and 1,438–1,441 in Pf-C3, respectively (**Figure 1**). The C3 convertase cleavage site of Pf-C3 was predicted as LDP (727–729) (**Figure 1**). The thioester motif GCGEQ (residues 1,006–1,010) and catalytical His- (residue 1,119) and Gln- (residue 1,121) in Pf-C3 are highly conserved among C3 homologs (**Figure 1**). Six highly conserved cysteine residues also existed in possible C3a region at residues 676, 677, 693, 709, 716, and 717 (**Figure 1**).

### Sequence Homology and Phylogenetic Analysis

To investigate the relationship of Pf-C3 with C3-like molecules identified from other vertebrates and invertebrates, the sequence homology and phylogenetic analysis were surveyed. Protein BLAST analysis yielded close matches of Pf-C3 with other C3 or C3-like molecules. The complete sequence of Pf-C3 exhibited a relatively high identity to C3 or C3-like proteins from mussel *Mytilus coruscus* (41.46% identity, GenBank accession No. AXS68445.1), *M. galloprovincialis* (41.46%, AJQ21542.1), scallop *Mizuhopecten yessoensis* (44.11%, OWF37722.1), *Pecten maximus* (44.77%, XP_033763816.1), and oyster *C. virginica* (48.99%, XP_022345651.1), *C. gigas* (49.11%, XP_034335787.1).

**Figure 2:**
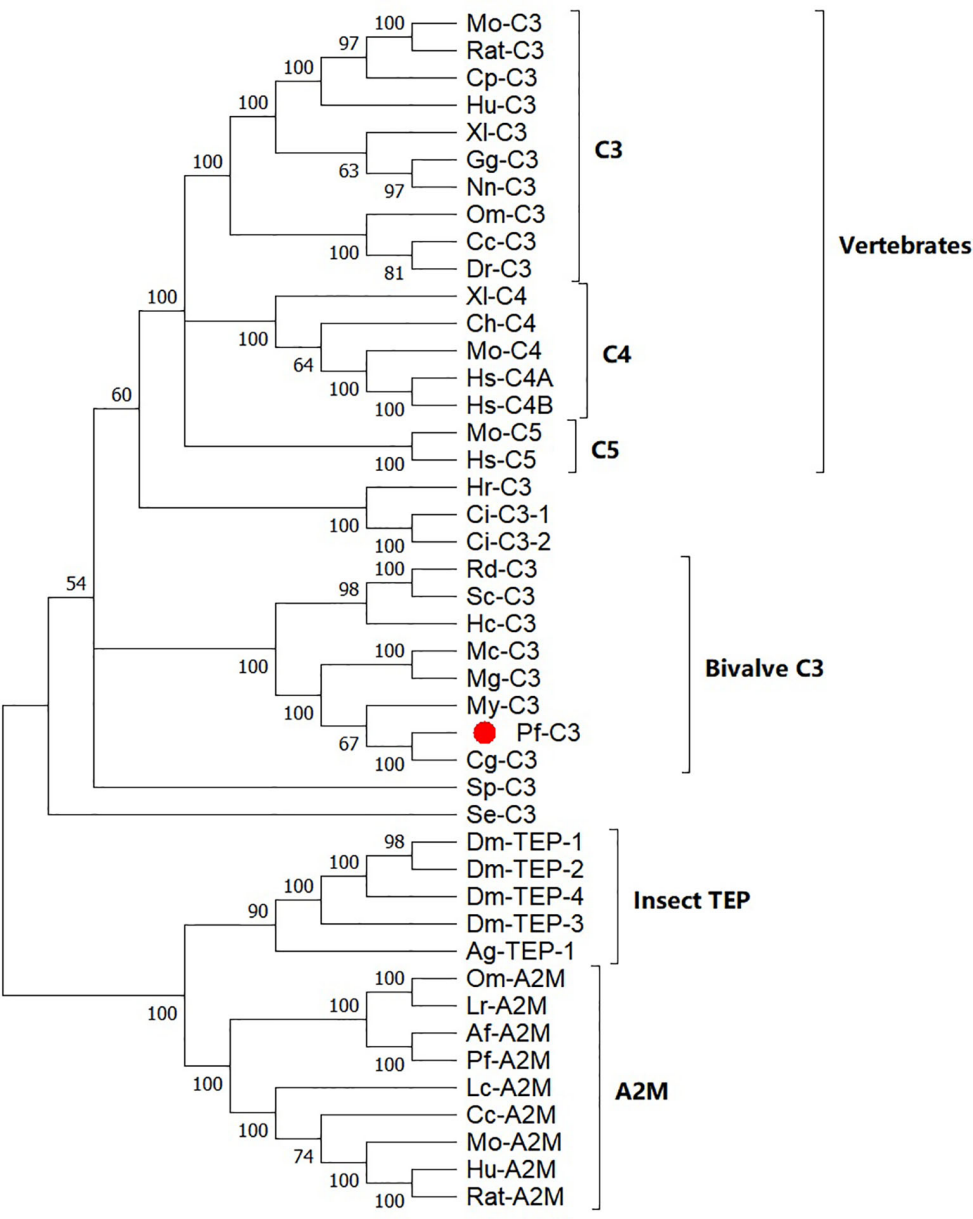
Phylogenetic analysis of *pf-C3* and other TEP family proteins with the Maximum Likelihood method. Sequences used to construct phylogenetic tree with the abbreviation and GenBank accession number are given below: Mo-C3, Mouse C3 (P01027.3); RatC3, Rat C3 (X52477.1); Cp-C3, *Cavia porcellus* C3 (P12387.2); Hu-C3, Human C3 (P01024.2); Xl-C3: *Xenopus laevis* C3 (AAB60608.1); Gg-C3, *Gallus gallus* C3 (Q90633); Nn-C3, *Naja naja* C3 (Q01833.1); DrC3, *Danio rerio* C3 (XP_696246.3); Om-C3, *Oncorhynchus mykiss* C3 (P98093.1); Cc-C3, *Cyprinus carpio* C3 (AB016210.1); Mo-C5, Mouse C5 (P06684.2); Hs-C5, Human C5 (P01031.4); Ch-C4, Chicken C4 (T28153); Xl-C4, *Xenopus laevis* C4 (BAA11188.1); Mo-C4, Mouse C4 (P01029.3); Hs-C4A, *Homo sapiens* C4A (AAB59537.1); Hs-C4B, *Homo sapiens* C4B (AAA99717.1); Hr-C3, *Halocynthia roretzi* C3 (AB006964.1); Ci-C3-1, *Ciona intestinalis* C3 (Q8WPD8); Ci-C3-2, *Ciona intestinalis* C3 (Q8WPD7); Rd-C3, *R. decussatus* C3 (ACN37845); Sc-C3, *Sinonovacula constricta* C3 (ANI85912); Hc-C3, *Hyriopsis cumingii* C3 (MK648113); Mc-C3, *Mytilus coruscus* C3 (MG197986); Mg-C3, *Mytilus galloprovincialis* C3 (AJQ21542); My-C3, *Mizuhopecten yessoensis* C3 (OWF37722); Cg-C3, *C. gigas* C3 (NP_001292308); Sp-C3, *Strongylocentrotus purpuratus* C3 (NP_999686); Se-C3, *Swiftia exserta* C3 (AAN86548); Dm-TEP-1, *Drosophila melanogaster* TEP-1 (CAB87807.1); Dm-TEP-2, (CAB87808.1); Dm-TEP-4, *D. melanogaster* TEP-4 (CAB87810.1); Dm-TEP-3, *D. melanogaster* TEP-4 (CAB87809.1); Ag-TEP-1, *Anopheles gambiae* TEP-1 (AF291654.1); Om-A2M, *Ornithodoros moubata* A2M (AAN10129); Ir-A2M, Ixodes ricinus A2M (EU835901.1); Af-A2M, *Azumapecten farreri* A2M (AAR39412.1); Pf-A2M, *Pinctada fucata* A2M (KF953540.2); Lc-A2M, *Lethenteron camtschaticum* A2M (D13567.1); Cc-A2M, *Cyprinus carpio* A2M (BAA85038.1); Mo-A2M, Mouse A2M (Q61838.3); Hu-A2M, Human A2M (P01023.3); Rat-A2M, Rat A2M (P06238.2).

Based on a multiple alignment of the amino acid sequences from TEP superfamily, a Maximum Likelihood (ML) phylogenetic tree was constructed to examine the phylogenetic relationships between mollusk C3s and other known C3 homologs (**Figure 2**). The resulting tree differentiated into two well-established clusters, one with the C3/4/5 components of the complement system including pf-C3, and another with the proteins from *α*2-macroglobuling (A2M) subfamily including A2Ms and insect TEPs. The complement group was divided into five subclades with C3s, C4s, and C5s from the vertebrates, sea squirt C3s, sea urchin C3, bivalve C3s, and coral C3. Pf-C3 was first clustered with C3 from *C. gigas* and following grouped with C3s from other bivalves.

### Tissue Expression Profile of *pf-C3*

**Figure 3:**
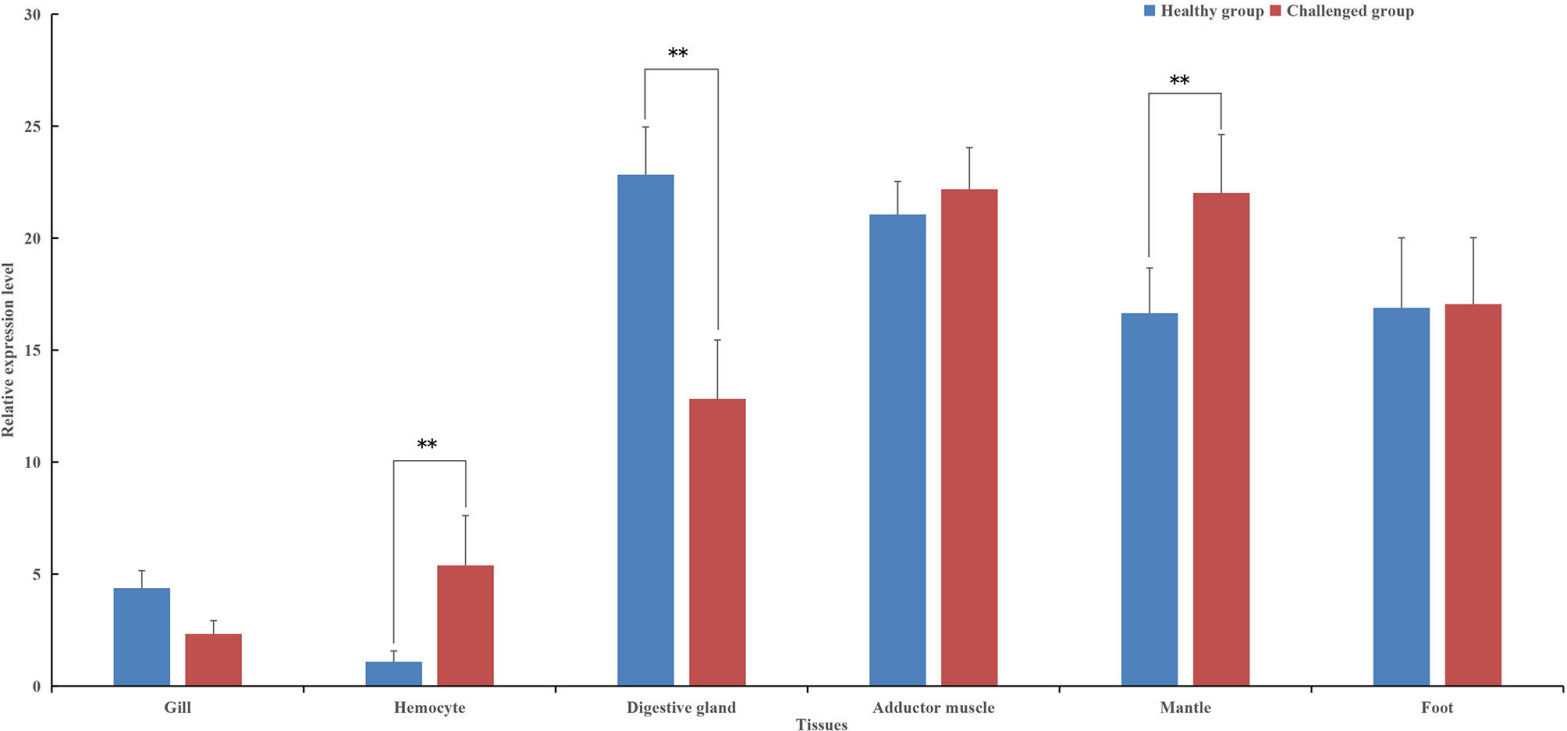
The tissue-specific expression of *pf-C3* was detected by Real-time PCR. Significant difference was indicated by asterisks, **P < 0.01.

The tissue-specific expression of *pf-C3* was also investigated by real-time PCR (**Figure 3**). *pf-C3* was ubiquitously distributed in all tested tissues with the highest level detected in the digestive gland, followed by the adductor muscle. After being challenged with *V. alginolyticus* 4* h* later, the expression level of *pf-C3* in hemocytes and mantle markedly increased, while at the same time, its expression in the digestive gland was down-regulated significantly, and the maximal expression level was found in the adductor muscle and mantle.

### Challenge-Induced Expression of *pf-C3* by *V. alginolyticus*

**Figure 4:**
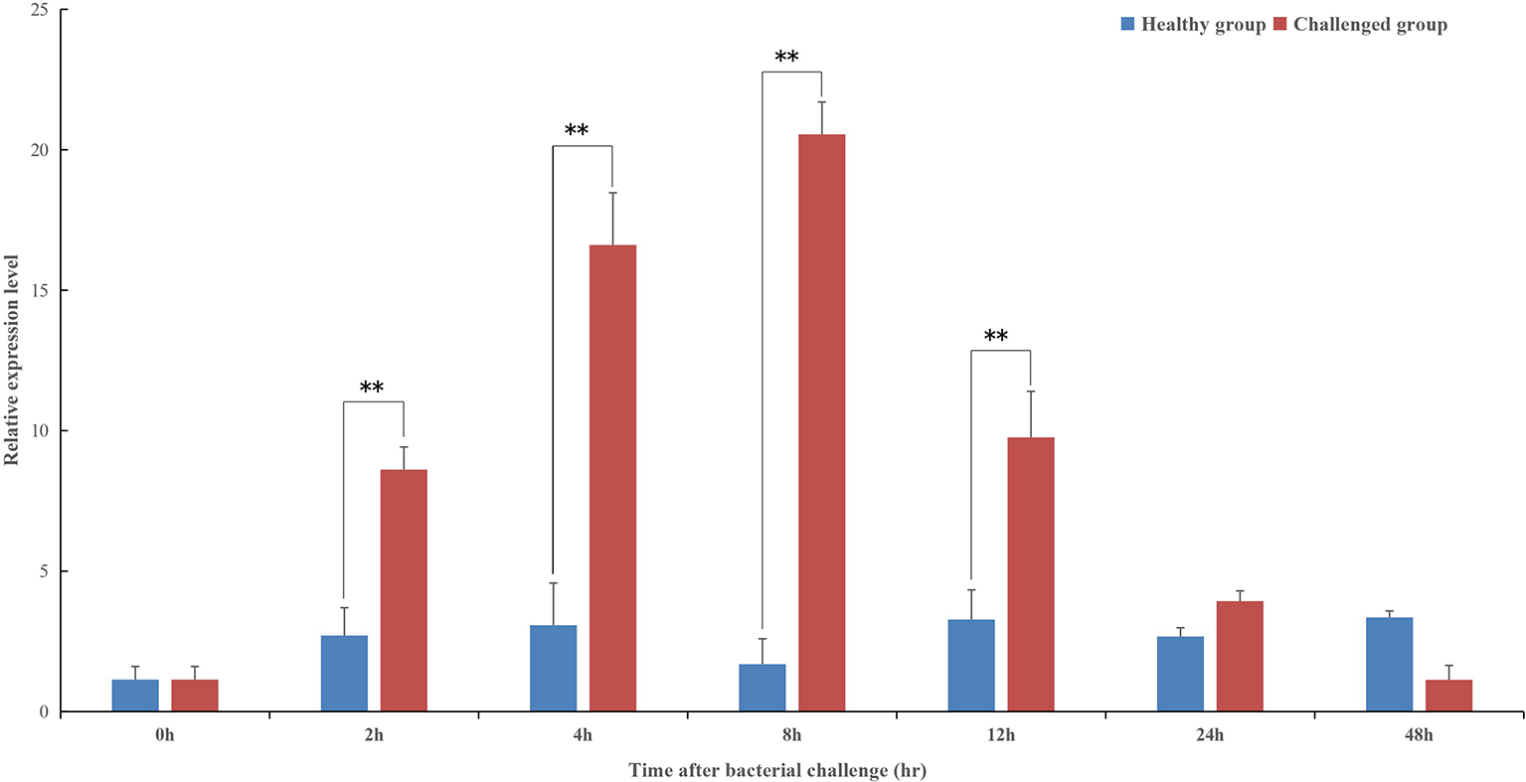
The temporal expression profiles of *pf-C3* in hemocytes of *P. fucata* challenged with *V. alginolyticus* were detected by Real-time PCR. Significant difference was indicated by asterisks, **P < 0.01.

The temporal expression profile of *pf-C3* in hemocytes of *P. fucata* challenged with *V. alginolyticus* was shown in **Figure 4**, and a clear time-dependent expression pattern was observed. At 2–4 h after bacterial challenge, the expression of *pf-C3* was up-regulated gradually and reached the maximum level at 8 h post-challenge. As time progresses, the *pf-C3* expression dropped at 12 h and recovered to the original level by 24 h post-challenge. Analysis of variance indicated that the expression levels of *pf-C3* mRNA at 2, 4, 8, and 12 h post-challenge were significantly higher than that of the control group (*P* < 0.05).

### Activation of NF-*κ*B Signaling Transduction by *pf-C3a*

**Figure 5:**
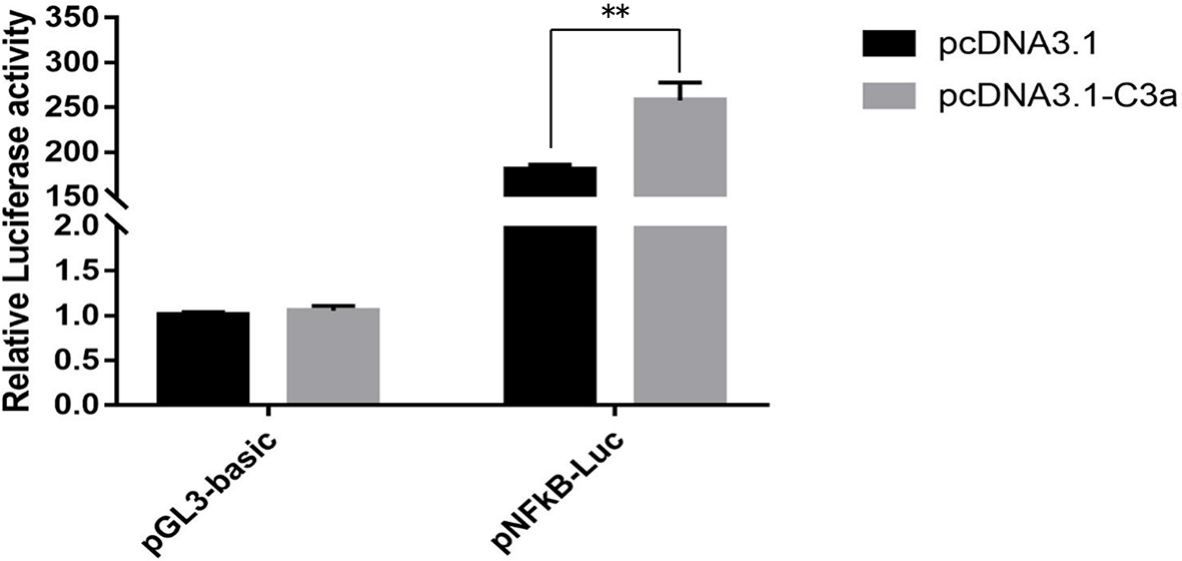
Dual-luciferase reporter assays of pNF-*κ*B activation in HEK293T transfected with *pf-C3a*. The bars indicated relative luciferase activity (n = 3). pcDNA3.1 and pGL3-Basic were used as the control. The amount relative to the internal control was expressed as mean ± S.D (n = 3). Significant differences across control were indicated (**P < 0.01).

To determine whether pf-C3a could promote the activation of immune signal pathway, dual-luciferase reporter assays were performed in this study. The results showed that expression of recombinant pf-C3a resulted in a 1.43-fold (*P* < 0.01) increase in pNF-*κ*B-Luc expression (**Figure 5**), which indicated pf-C3a could activate the expression of luciferase reporter genes, suggesting that it could promote transcription of vertebrate target genes containing the NF-*κ*B binding site in HEK293T cells, that is to say, pf-C3a involved and activated NF-*κ*B signal pathway *in vitro*.

### Knockdown of *pf-C3* by siRNA

**Figure 6:**
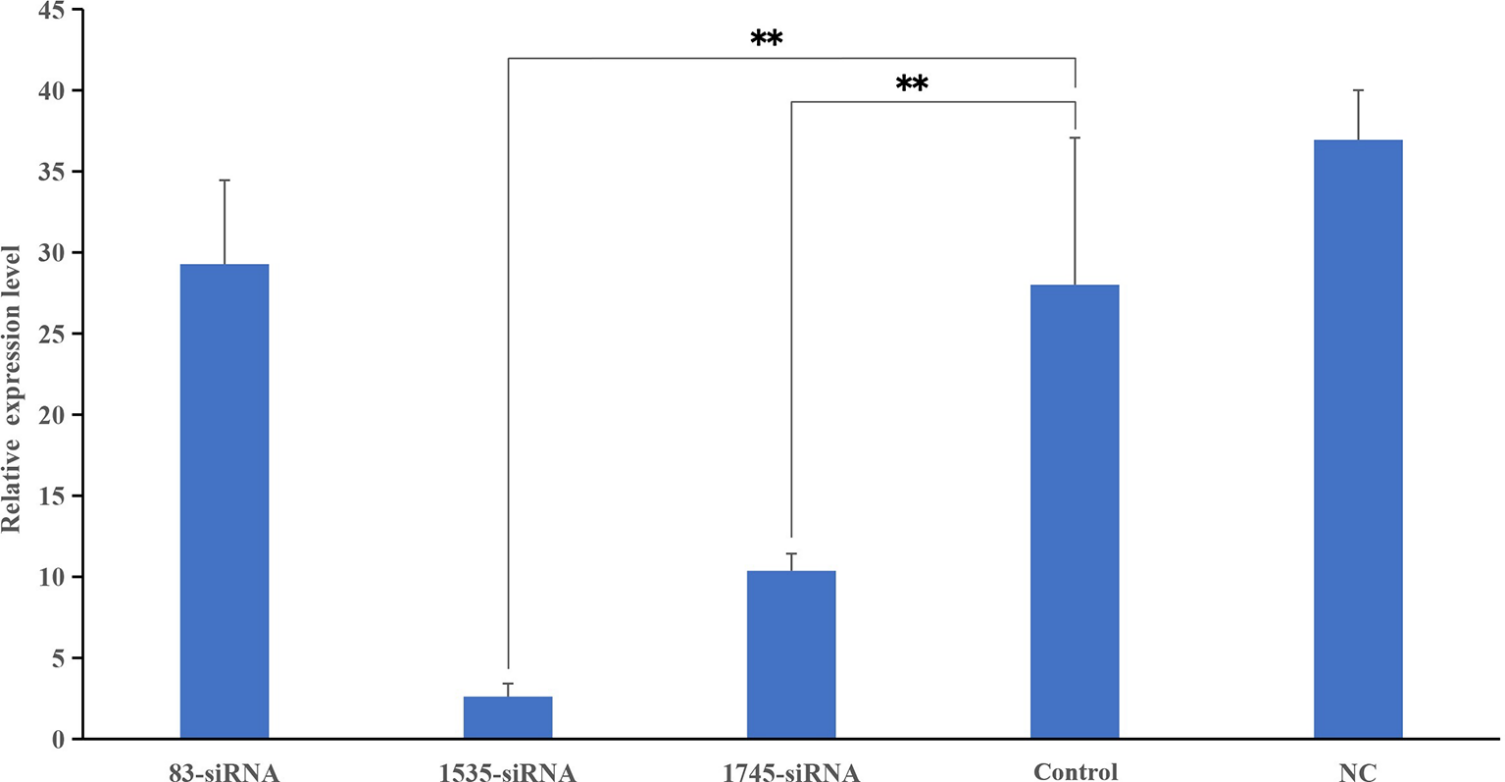
Knockdown efficiency detected by Real-time PCR. The relative expression levels of *pf-C3* after RNA interference, PBS as control. Significant difference was indicated by asterisks, **P < 0.01.

Three different siRNAs were synthesized and injected into healthy *P. fucata*, while *P. fucata* in the control group were injected with an equal volume of PBS. After 24 h, the hemolymph of diverse groups was collected, and the relative gene expression levels between the test and control tissues were detected. As shown in **Figure 6**, two siRNAs significantly inhibited the expression level of *pf-C3*, and 1535-siRNA showed the best inhibitory effect compared with the control treatment.

### siRNA-Mediated Knockdown of *pf-C3* Reduces the Phagocytosis of *V. alginolyticus*

**Figure 7:**
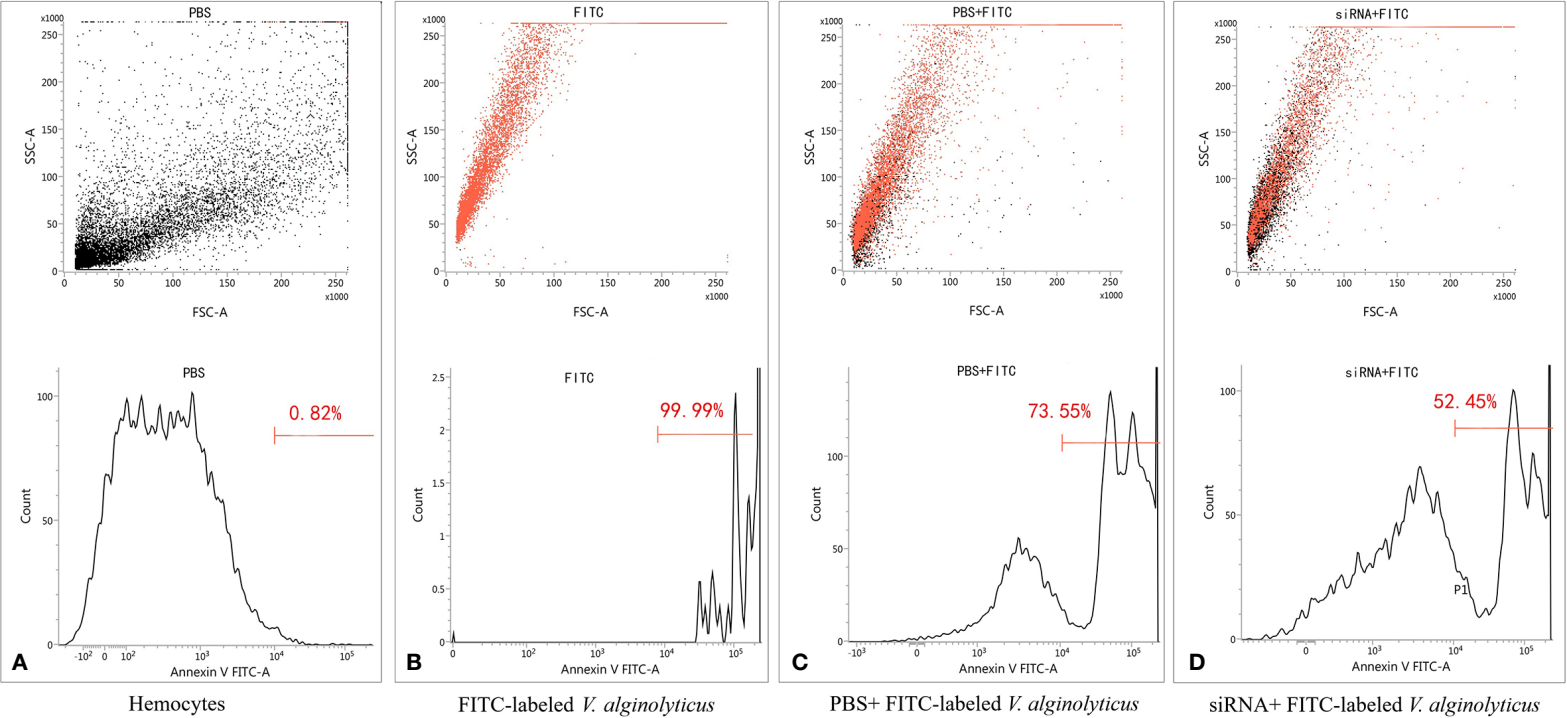
Flow cytometry assay of phagocytosis. **(A)** Hemocytes. **(B)** FITC-labeled *V. alginolyticus*. **(C)** PBS+ FITC-labeled *V. alginolyticus* **(D)** siRNA+ FITC-labeled *V. alginolyticus*.

To address the relationship between *pf-C3* expression and hemocyte phagocytosis, the *in vitro* phagocytosis assay was performed through flow cytometry analysis (**Figure 7**), and the result revealed that knockdown of *pf-C3* significantly lowered the phagocytosis of *V. alginolyticus* from 73.55 to 52.45% compared with the control, which means fewer *V. alginolyticus* could be engulfed by hemocytes treated by siRNA.

## Discussion

As invertebrates, mollusks lack an adaptive immune system but have developed various innate immune components and reactions including a set of humoral and cellular immune reactions in dealing with a diverse array of pathogens and stress responses (26). The innate immune responses in mollusks have been well described in several species; however, the exploration of genomic and transcriptomic data is providing new insights regarding several aspects of invertebrate immunology (4). In the present study, we determined the full-length cDNA of C3 (designated *pf-C3*) from the pearl oyster *P. fucata* and demonstrated that pf-C3 was involved in the activation of NF-*κ*B signal pathway *in vitro*, and knockdown of *pf-C3* by specific siRNA could significantly reduce the phagocytosis of *V. alginolyticus* by hemocytes.

As a highly conserved gene, the structure of C3 in invertebrates is considered to contain eight domains, according to Nonaka (27). In the pf-C3 deduced amino acid sequence, these C3-specific domains were also detected, including the *α*2M domains, such as A2M_N, A2M_N_2, ANATO, A2M, A2M_recep, and A2M_comp, and complement-specific domains, such as C345C (**Figure 1**). These recognitions of the conserved motifs identified pf-C3 as one member of C3 family and suggested that the pf-C3 possesses the basically physiological functions which are conserved in C3 throughout evolution (3).

The multiple alignment of pf-C3 with other bivalve C3s revealed two putative cleavage locations, conserved *β*–*α* junction site (RDRR) and *α*–*γ* junction site (RVGR), which predicted matured Pf-C3 shared the constitution of three chains after post-translational processing (**Figure 1**). However, the sequence for *β*–*α* junction site varies among C3 molecules. In bivalves, for example, the putative cleavage sequence RDRR occurs in *P. fucata*; RGKR occurs in *M. yessoensis*; RKRR occurs in *C. gigas*, and *Ruditapes decussatus*; and RRKR occurs in the C3 molecules of *M. coruscus*, *M. galloprovincialis*, *Sinonovacula constricta*, and *Hyriopsis cumingii* (**Supplementary Figure 2**) (19). Thus, alignment of the currently available bivalve C3 sequences suggests that RXXR is the consensus sequence for the *β*–*α* cleavage site (X is frequently R or K). For the linker between the *α* and *γ* chains in pf-C3, it was also observed in other invertebrate C3s, such as amphioxus (11), sea cucumber (15), coral (16), sea anemone (1), and bivalves (**Supplementary Figure 2**), including *M. yessoensis*, *M. coruscus*, *M. galloprovincialis*, *R. decussatus*, *S. constricta*, and *H. cumingii* and is considered to be a characteristic of ancestral C3; as the vertebrate C3, and C5 apparently lost this entire region, only C4 retained a part of it in vertebrates, which is used as the *α*–*γ* processing signal (15).

In addition to the presence of putative cleavage sites, the presence of a putative anaphylatoxin (ANATO) domain (residues 676–717, **Figure 1**) provided further support for the post-translational maturation of the pf-C3. The ANATO domain, a conserved character among complement molecules, is located at the N-terminus of the *α* chain. Sequence analysis of the putative pf-C3 ANATO domain revealed that it contains, in conserved locations, the six canonical cysteine residues known to be critical for its function (11) (**Figure 1**). Additionally, a putative cleavage site (LDP, residues 727–729) for the C3-convertase, which releases the ANATO domain and thereby creates C3b, was identified, and this cleavage site was once considered to be strictly conserved in most animals as LXR; however, it has been recently demonstrated not well conserved in invertebrates, as reported for *Apostichopus japonicus*, *Swiftia exserta* and *Haliplanella lineate* RXR, *Halocynthia roretzi* TSR, *M. coruscus* and *M. galloprovincialis* FDR, and *R. decussatus* LDD (15, 16, 28, 29) (**Supplementary Figure 2**).

The thioester region (GCGEQ) in pf-C3 is the most conserved and important aspect of the C3 amino acid sequence, and it is believed that the thioester bond is formed between Cys- and Gln- at the thioester motif bond in native C3 (**Figure 1**). Meanwhile, the conservative His-1108 and Gln-1110 residues (**Figure 1**), which are generally depicted to be the key regions guiding C3 fix itself on the surface of xenobiotics (30), were found in pf-C3, and detection of these catalytical residues indicate that pf-C3 may possess the substrate binding specificity in thioester (10).

According to the phylogenetic analysis, A2Ms, insect TEPs, and C3/4/5 molecules in the complement system could be distinguished and showed similar phylogenetic distribution to previously published trees of TEP superfamily (2, 16, 19, 21, 31–33) (**Figure 2**). Although the bootstrap valves at some nodes were less than 70, and the resolution of the phylogenetic tree was not sufficient to establish with certainty the phylogenetic relationships between bivalve C3s, echinoderm/urochordate C3s, and vertebrate C3/C4/C5, the separation of the phylogenetic clusters, and the initial BLAST results conclusively identified pf-C3 that belonged to the C3 subfamily, rather than the A2M subfamily. C3s form different taxonomic groups of invertebrates formed the clades of the ancestral C3 proteins, and no sequences clustered with the C4s and C5s from vertebrates suggested that C3 may have diverged earlier, and as a result, has characteristics of different complement proteins from high vertebrates that evolved later, and vertebrates C4 and C5 may have evolved from a C3-like molecule (4, 19, 34). Furthermore, the presence of both C3 and A2M in the *P. fucata* not only deepened the notion of C3 occurrence in protostomes, but also strengthened the certainty of the possession of both C3 and A2M in mollusk (21). Previously, it has been suggested that protostomes experienced differentially the loss of C3 and A2M during TEPs evolution, since no genes of C3 or A2M were found in the genomic sequencing data of many arthropods (21, 35), while now, it appears that the loss of some TEPs may only happen in some specific Arthropoda lineages (21).

In humans, the main organ of C3 synthesis is the liver (36), and the putative homologous organ in *P. fucata* is the digestive gland (4), which has a constitutive expression level of 20-fold higher when compared with hemocytes (**Figure 3**). This is consistent with general understanding of C3 synthesis in mammalian studies (35, 37, 38). Hemocytes are fundamental immunocytes responsible for the recognition and elimination of infected pathogens in mollusks. In this study, the expression of pf-C3 significantly increased in hemocytes of *P. fucata* at 4 h after *V. alginolyticus* challenge, and reached the peak level at 8 h (12-fold increases, *P* < 0.01) (**Figure 4**), which indicated that pf-C3 possibly played a role of immune modulation in the acute-phase response to bacterial challenge in *P. fucata*, and might function as a pattern recognition molecule in the innate immune system.

C3 is well recognized as the central mediator in the complement cascade, whose activation leads to the production of C3a and C3b fragments. To date, however, studies focused on complement-mediated immune processes elicited by the anaphylatoxin C3a-like molecules in invertebrates are still fragment. Ascidian C3a-like peptides have been demonstrated to stimulate chemotaxis by hemocytes from *Pyura stolonifera* (39) and *C. intestinalis* (40). Additionally, the activated C3a from the invertebrate chordate *Branchiostoma japonicum* was shown to be capable of inducing vertebrate macrophage migration and enhancing macrophage phagocytosis and respiratory burst response. Meanwhile, in terms of proinflammatory factors, C3a mediates the expression of many inflammatory cytokines, including those associated with NF-*κ*B pathway, which is essential for the host defense system and responsible for the regulation of inflammatory cytokine expression responding to injury or infection (41–43). To determine the involvement of *pf-C3a* in the activation of NF-*κ*B signal pathway, dual-luciferase reporter assays were performed in the present study (**Figure 5**), and the results showed that the luciferase reporter gene pNF-*κ*B-Luc was activated significantly by overexpression of pf-C3a recombinant plasmid, revealing that pf-C3a involved in the regulation of vertebrate target genes containing the NF-*κ*B binding site in HEK293T cells. These data implied that C3a mediated the activation of NF-*κ*B may be conserved throughout evolution (3).

In mammals, the phagocytic activity of neutrophils and macrophages is elevated through the activation of the complement system, which generates a number of fragments that bind to target cells, and deposition of C3 is a critical prerequisite for phagocytosis (44). Interaction of the opsonized cells with C3 receptors on the phagocytes results in an enhancement of the phagocytosis. For invertebrates, phagocytic cells are the first line of defense of the innate immune system (45). Phagocytosis is a vital process including recognition of pathogens, assembly of cell membrane, maturation of phagosomes, and digestion of the particle in the phagosome (46). In this study, siRNA-mediated knockdown of *pf-C3* resulting in significant reduction of *V. alginolyticus* phagocytosis by hemocytes indicated that pf-C3 participated in the phagocytosis process and positively influenced phagocytic cells (**Figure 7**). Moreover, after being challenged by *V. alginolyticus*, pf-C3 may be activated and cleaved into C3b, which is attached to the surface of target cells in the cytolytic process. Further studies are still needed to increase our understanding of the mechanism by which pf-C3 fulfills its function as an immune molecule in the immune system of *P. fucata*.

In conclusion, the cDNA cloning and characterization of a complement component 3 (*pf-C3*) from *P. fucata* were performed in this work. Both the highly conserved structural features and results of phylogenetic analysis ascertained pf-C3 as a typical C3 homolog. The *pf-C3* mRNA expressed highly in the digestive gland, adductor muscle, foot and mantle, and its expression was significantly increased in the hemocytes after *V. alginolyticus* challenge. Dual-luciferase reporter assays showed that pf-C3a could trigger the activation of NF-*κ*B signaling transduction. Furthermore, knockdown of *pf-C3* by specific siRNA could significantly reduce the phagocytosis of *V. alginolyticus* by hemocytes *in vitro*. The results in this study would help increase understanding of the function of C3 in the invertebrate immune system and therefore provide new insights into the roles of the primitive complement system in invertebrates.

## Data Availability Statement

The datasets presented in this study can be found in online repositories. The names of the repository/repositories and accession number(s) can be found in the article/**Supplementary Material**.

## Author Contributions

ZW and JW wrote the manuscript. All authors contributed to the article and approved the submitted version.

## Funding

This work was supported by grants from The Natural Science Foundation of Guangdong Province, China (2019A1515011875), International Science and Technology Cooperation Project of Science and Technology Planning Project of Guangdong Province, China (2019A050510044), National College Student Innovation and Entrepreneurship Training Program, (201810566001, CXXL2020002).

## Conflict of Interest

The authors declare that the research was conducted in the absence of any commercial or financial relationships that could be construed as a potential conflict of interest.
