# Supplementary material for: Molecular Characterization of Complement Component 3 (C3) in the Pearl Oyster Pinctada fucata Improves Our Understanding of the Primitive Complement System in Bivalve
Source: Front Immunol. 2021 Apr 19;12:652805. doi: 10.3389/fimmu.2021.652805 (PMC8089394; doi:10.3389/fimmu.2021.652805)
Supplement: Supplementary file 1 [file DataSheet_1.docx]

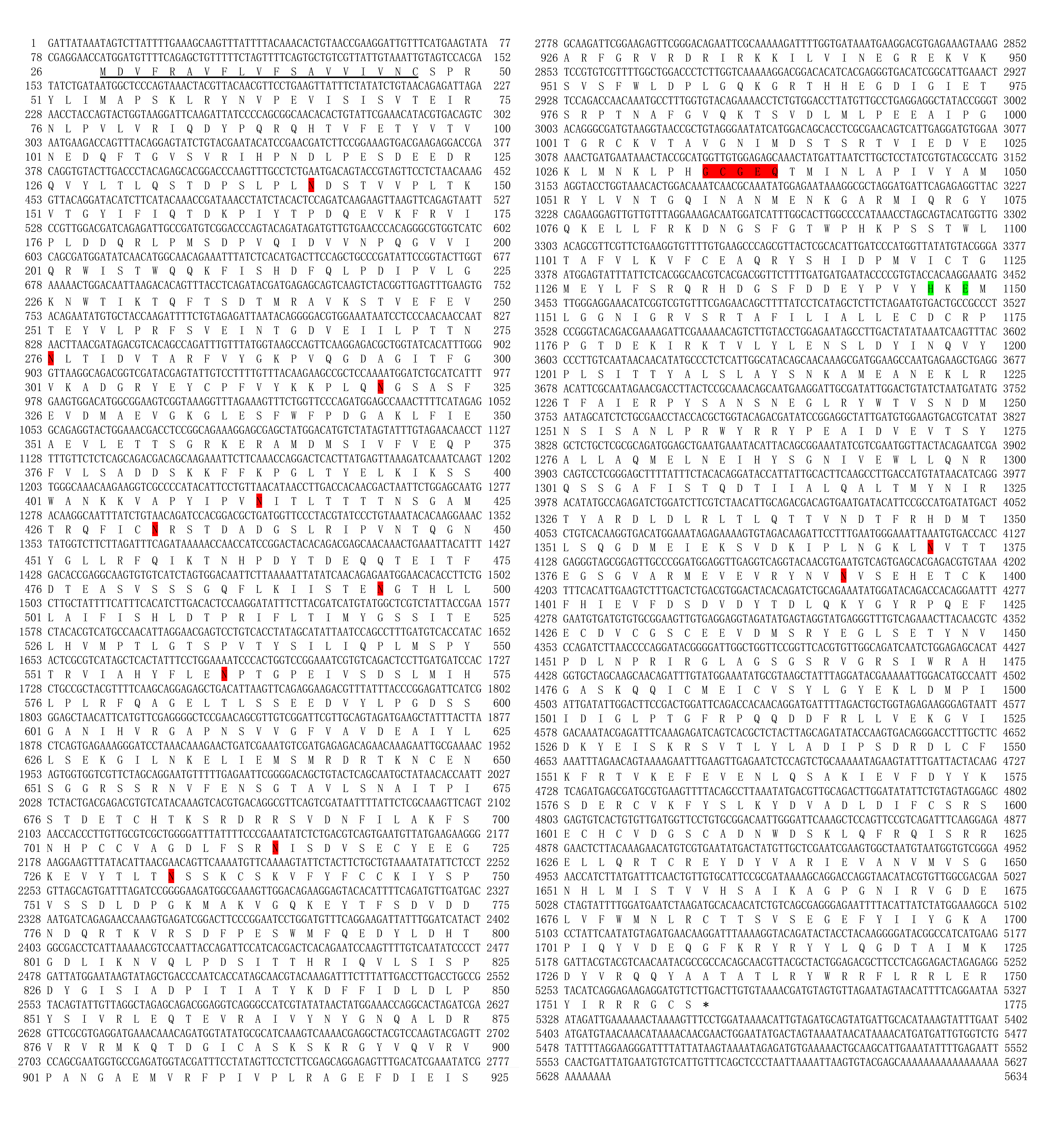


**Supplementary Figure 1.** The complete nucleotide and deduced amino acid sequence of pf-C3 from *P. fucata*. The putative sequence of signal peptide is underlined with black single line. The glycosylation sites and thioester motif are marked in red. The catalytical His and Gln are marked in green.
