# Supplementary material for: Molecular Characterization of Complement Component 3 (C3) in the Pearl Oyster Pinctada fucata Improves Our Understanding of the Primitive Complement System in Bivalve
Source: Front Immunol. 2021 Apr 19;12:652805. doi: 10.3389/fimmu.2021.652805 (PMC8089394; doi:10.3389/fimmu.2021.652805)
Supplement: Supplementary file 2 [file DataSheet_2.docx]

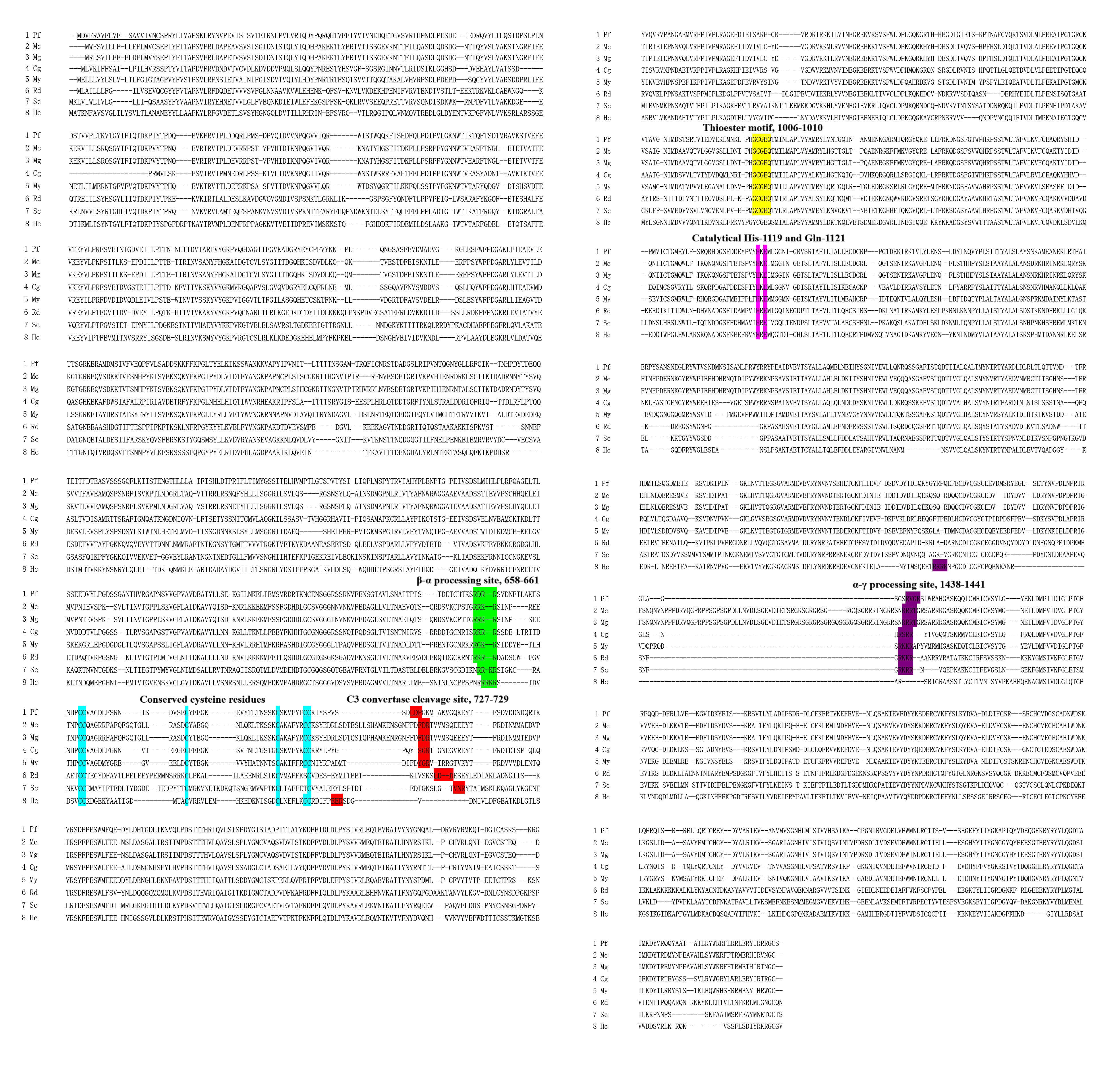


**Supplementary Figure 2.** Multiple alignments of amino acid sequences of pf-C3 with other bivalve C3s. β-α processing site is marked in green, α-γ processing site is marked in purple, C3 convertase cleavage site is marked in red, thioester motif is marked in yellow, conserved Cys is marked in blue, catalytical His and Glu residues are marked in pink. The abbreviations of species names and GenBank accession number are: Pf, *Pinctada fucata* C3 (MT502525); Mc, *Mytilus coruscus* C3 (MG197986); Mg, *Mytilus galloprovincialis* C3 (AJQ21542); Cg, *Crassostrea gigas* C3 (NP_001292308); My, *Mizuhopecten yessoensis* C3 (OWF37722); Rd, *Ruditapes decussatus* C3 (ACN37845); Sc, *Sinonovacula constricta* C3 (ANI85912); Hc, *Hyriopsis cumingii* C3 (MK648113).
